# Impact of the COVID-19 pandemic on consultation-liaison activity: changes in everyday clinical practice and work-related factors associated with the psychiatrist’s need for seeking help

**DOI:** 10.1192/j.eurpsy.2023.2456

**Published:** 2023-10-26

**Authors:** Giorgio Mattei, Krzysztof Krysta, Massimo Clerici, Fabrizia Colmegna, Vladimir Korostiy, Dalibor Karlović, Marek Krzystanek, Gilbert M.D. Lemmens, Pieter-Jan Geerts, Filipa Novais, Vjekoslav Peitl, Jacopo Santambrogio, Dan Georgescu, Diogo Telles Correia

**Affiliations:** 1 Association for Research in Psychiatry, Castelnuovo Rangone, Modena, Italy; 2 Department of Rehabilitation Psychiatry, Medical University of Silesia, Katowice, Poland; 3 Department of Medicine and Surgery, University of Milano-Bicocca, Milan, Italy; 4 Consultation-Liaison Psychiatry Service, Department of Mental Health and Addiction (DSMD), ASST Monza, Monza, Italy; 5 Department of Psychiatry, Narcology and Medical Psychology, Kharkiv National Medical University, Kharkiv, Ukraine; 6 Department of Psychiatry, University Hospital Center Sestre Milosrdnice, Zagreb, Croatia; 7 School of Medicine, Catholic University of Croatia, Zagreb, Croatia; 8 Department of Rehabilitation Psychiatry, Medical University of Silesia, Katowice, Poland; 9 Department of Psychiatry, Ghent University Hospital, Ghent, Belgium; 10 Department of Head and Skin – Psychiatry, Ghent University, Ghent Belgium; 11 University of Lisbon, Lisbon, Portugal; 12 Department of Psychiatry, AZ Groeninge, Courtrai, Belgium; 13 Adele Bonolis AS.FRA. Foundation, Vedano al Lambro, Italy; 14 Presidio Corberi, ASST Brianza, Limbiate, Italy; 15 Department of Consultation-Liaison Psychiatry, Old Age Psychiatry and Neurodevelopmental Psychiatry, Psychiatric Services Aargau, Windisch, Switzerland

**Keywords:** burnout, consultation-liaison psychiatry, COVID-19, psychosomatics, work-related factors

## Abstract

**Background:**

Since the very beginning of the COVID-19 pandemic, consultation-liaison psychiatry (CLP) services were in first line to face the effects of the novel virus.

**Aim:**

Aim of this study was to assess the activity of CLP services during the pandemic, and to identify specific work features associated with the need for psychological/psychiatric support by the CL psychiatrist during the pandemic.

**Methods:**

This study was carried out in six European countries. A questionnaire was developed to collect information concerning psychiatric referrals for patients affected and not affected by COVID-19. Multivariate binary logistic regressions were used to study work-related factors associated with the need for psychological and/or psychiatric support by the CL psychiatrist during the pandemic.

**Results:**

The sample included 108 psychiatrists working in CLP services in six countries. The majority reported that the pandemic had not impacted on their work with non-COVID-19 patients. Among patients affected by COVID-19, the most common psychiatric diagnoses were anxious-depressive disorders related to COVID-19, delirium due to COVID-19, anxious-depressive disorders unrelated to COVID-19, suicidal ideation related to COVID-19. The 25% of psychiatrists reported having needed psychological or psychiatric support during the pandemic due to assisting COVID-19-positive patient. The need for support was associated with increased age, few years of medical experience and experience of anxiety while consulting COVID-19-positive patients. The risk was reduced using personal protective equipment and teleconsulting.

**Conclusions:**

Findings prompt to the need of addressing health issues of health care professionals, namely psychiatrists working in the field of CLP and psychosomatics.

## Introduction

On March 11, 2020, the World Health Organization (WHO) declared the novel corona virus-19 disease (COVID-19) a pandemic, the virus being present in 160 nations and in all continents [1]. Since then, the world has been experiencing an unprecedented public health crisis, with consequences not only for physical health but also for the mental health of the global population [2].

Italy has been the first democracy and the first European country that faced the epidemic, with the first case reported on February 21, 2020 [3–5]. In the following weeks, the virus quickly spread throughout the other European Member States, leading to a condition of uncertainty, fostered by the fear to be infected, as well as to infect other people [6]. While the virus was spreading throughout the rest of Europe, the first cases were reported in the United States, involving health care professionals, that represented a particularly vulnerable group, from the onset of the pandemic [7]. In the beginning of April 2020, over 9,000 health care professionals were infected by COVID-19 in the United States, and at least 27 were killed [8].

Since the very beginning of the pandemic, consultation-liaison psychiatry (CLP) services were in first line to face the effects of the novel virus [9–11]. As it is known, CLP is placed at the convergence of psychiatry and the rest of medical specialties [8]. CLP (also known as psychiatry of the medically ill, liaison psychiatry, or psychosomatics) is a sub-specialty of psychiatry dealing with the interface between somatic and psychological disciplines. It is framed within the bio-psychosocial paradigm, which aims to a holistic approach to illness, rather than a biomedical one [12, 13].

Because of the onset of the COVID-19 pandemic, CLP services had to adjust their work and organization, shifting to a hybrid model which means working remotely when possible and carrying out face-to-face consultations when necessary [8]. The hybrid model has training implications for residents as well [8, 14].

After the outbreak of the pandemic, several studies investigated its potential impact on health care professionals. Guillen-Burgos et al. [15] reported increased mental health outcomes in this group of workers, with the most at-risk work environment represented by intensive care units (ICUs) and emergency room. Stafseth et al. [16] investigated ICU workers in Norway and found that most of them felt socially isolated and reported a fear of infecting others. More than one respondent out of 10 (both nurses and physicians) were potentially affected by anxious-depressive disorders. Symptoms suggesting the presence of post-traumatic stress disorder (PTSD) were frequent as well. Features associated with increased risk of distress were younger age and less than 5 years of previous work experiences.

Research focused also on the differences between first- and second-line healthcare workers during the COVID-19 pandemic. Among first-line workers, extraordinary levels of stress were reported from the very beginning of the pandemic, possibly due to increased case numbers and working hours [17]. High rates of psychiatric symptoms, namely PTSDs symptoms, among front-line health care workers were reported in China [18], Italy [19, 20], Oman [21], United States [22, 23], and South Africa [24]. A study carried out in Italy showed that frontline workers had higher prevalence of insomnia, depression, anxiety, obsessive–compulsive symptoms, non-specific chronic and acute traumatic stress, as well as more adaptive coping strategies, when compared with second-line workers [20].

With respect to the impact of the pandemic on CLP, research adopting an international view is scant [25]. Moreover, to the best of our knowledge, no study focused on work-related factors associated with consultations-liaison psychiatrists’ need for seeking help because of the pandemic. Therefore, aim of this study was to describe the activity of CLP services throughout Europe during the COVID-19 pandemic and to identify specific work features associated with the psychiatrists’ need of psychological/psychiatric support while assisting COVID-19-positive patients during the pandemic. Building on existing literature, our research hypothesis was that the COVID-19 pandemic may have caused psychopathological symptoms among consultants, and, at organizational level, increased requests for referral to the CLP services.

## Methods

### Study design

This is a cross-sectional study. The complete research protocol is available at the following link: https://www.europsy.net/sections/?id=3.

An ad hoc questionnaire was developed to collect sociodemographic data and information concerning psychiatric referrals requested for patients affected and not affected by COVID-19. Data were statistically analyzed. The questionnaire had the following structure. In the first part, socio-demographic information was collected. In the second part, questions concerning working with non-COVID-19 patients were asked. In the third part, questions concerning working with COVID-19 patients were asked.

To identify consultation-liaison psychiatrists potentially eligible, a snowball methodology was used. First, all members of the EPA section on CLPP received an invitation to participate to the study via e-mail, with the ad hoc questionnaire attached. Besides being asked to participate to the study, CLP psychiatrist were asked to share the invitation with their colleagues working in the CLP field, irrespectively from their membership to the EPA.

All questionnaires were anonymized. Descriptive and inferential statistics were carried out using STATA 12.1. The guidelines governing research from the Declaration of Helsinki were followed.

As far as descriptive statistics is concerned, means, standard deviations, and frequencies were used when appropriate. Specifically, when visual inspection pointed out a normal distribution of the variable assessed, means and standard deviation were used.

With respect to inferential statistics, univariate and multivariate logistic regressions were used. In all regressions, the outcome was a binary variable suggesting the need for psychological and/or psychiatric support by the CL psychiatrist during his/her work during the pandemic, and specifically while assessing patients affected by COVID-19. The new variable was created according to the responses given to the item 16 of the questionnaire “Did you need a psychological/psychiatric support in your work during this period (or after it) due to your assistance COVID-19-positive patients?” Possible answers: yes, no, in some cases. The new binary variable was equal to 0 when the answer to item 16 was “no” and was equal to 1 otherwise.

First, all variables included in the three parts of the questionnaire were individually tested, using as outcome the new variable described above. All variables that reached a significance level indicated by a *p*-value <0.20 were included in the multivariate logistic regressions. This significance cut-off, higher than the usual one, was set to reduce type II error, that is, the possibility to exclude potentially significant variables from the final analysis [26].

Second, three stepwise multivariate logistic regressions were built, one per each part of the questionnaire. The first multivariate regression dealt with socio-demographics, explored in the first part of the questionnaire. The second multivariate regression dealt with data concerning working with non-COVID-19 patients, explored in the second part of the questionnaire. Finally, the third multivariate regression dealt with data concerning working with COVID-19 patients, explored in the third part of the questionnaire. In all multivariate regressions, the usual level of significance indicated by a *p*-value <0.05 was adopted.

## Results

### First part of the questionnaire (socio-demographic information)

One hundred and eight questionnaires were filled in and available for data analysis. Respondents were from the following countries (*N*; %): Belgium (22; 20%), Croatia (22; 20%), Portugal (21; 19.5%), Italy (20; 18.5%), Ukraine (13; 12.5%), and Poland (10; 9.5%). All data came from University Hospitals, except Ukraine, where data came from a University Hospital (13.4%) and a second-level hospital (86.6%). Mean age was 42 ± 11. Fifty-three respondents were women (49% of the sample), mostly specialists (*N* = 81, 75%). Twenty-seven residents were enrolled (25% of the sample). Mean years of medical experience were 16 ± 11. Fifty-four respondents (61% of the sample) were members of national/international psychiatric or medical associations, including the EPA.

### Second part of the questionnaire (items involving non-COVID-19 patients)

The second part of the questionnaire investigated referrals involving patients not affected by COVID-19, as displayed in Table 1.

**Table 1.** Responses to the second part of the questionnaire, concerning consultation-liaison activity with non-COVID-19 patients

Item	Responses	*N* (%)
In which conditions do you carry out referrals? Please tick one or more options	Face-to-face consultations using protective face mask	91 (85)
	Face-to-face consultations using a protective uniform	29 (27)
	Consultations based on the interview with the ward doctor responsible for the patient	28 (26)
	Consultations using the internal phone line in the hospital	21 (20)
	Consultations using the patient’s mobile phone	18 (17)
Did the COVID-19 pandemic have an impact on the method you use to visit hospitalized non-COVID-19 patients?	Yes	42 (42)
	No	58 (58)
Did the requests of referral for non-COVID-19 patients change during this period?	No	38 (38)
	They were less	28 (28)
	They were more	34 (34)
Did the diagnosis differ in non-COVID-19 patients during this period?	Yes	10 (10)
	No	90 (90)
Did you see COVID-19 hospitalized patients?	Yes	90 (89)
	No (If NO, the questionnaire stops)	11 (11)

Forty-two respondents reported that the COVID-19 pandemic had an impact on the method they use to visit hospitalized non-COVID-19 patients. When asked to specify, they reported the following answers (*N*): use of protective face mask (4 respondents); interviews with safety distance (4); careful use of psychometric instruments; impact on the differential diagnosis; less contact with family members (and less visits from them in the hospital); less multidisciplinary clinical meetings concerning hospitalized patients; more time needed for the examination; more scheduled admissions; mandatory screenings, that is PCR and rapid antigen test (3); impact on group therapies (i.e., groups were smaller or interrupted) (2); impact on therapeutic possibilities (2); no experience in psychiatry before COVID-19; wearing protective equipment; important limitations in the observations of expressivity and mimicry that affects the quality of clinical interview; more attention to protective precaution; before visiting a patient, temperature is taken and some COVID-19-related questions are asked; more telephone consultations; fewer face-to-face consultations; more anxiety while consulting; change in the clinical manifestation of typical features of a disease; responsibility; shorter consultations.

When the answer to the last question reported in Table 1 (Did you see COVID-19 hospitalized patients?) was “no,” the questionnaire stopped. Differently, when the answer was “yes,” a third part was available to fill in.

### Third part of the questionnaire (items involving COVID-19 patients)

Table 2 presents the results concerning the third part of the questionnaire, which investigated clinical CLP activity with patients affected by COVID-19.

**Table 2.** Responses to the second part of the questionnaire, concerning consultation-liaison activity with COVID-19 patients

Item	Responses	*N* (%)
Did you have any specialized training in the treatment of patients affected by COVID-19?	Yes	16 (20)
	No	66 (80)
In which conditions do you carry out referrals with patients affected by COVID-19? Please tick one or more options	Face-to-face consultations using a protective uniform	68 (80)
	Face-to-face consultations using protective face mask	37 (44)
	Consultations based on the interview with the ward doctor responsible for the patient	29 (34)
	Consultations using the internal phone line in the hospital	24 (28)
	Consultations using the patient’s mobile phone	13 (15)
Which are the most common psychiatric diagnoses in this group of patients?	Anxious-depressive disorders *related* to COVID-19	54 (64)
	Delirium *due to* COVID-19	36 (42)
	Anxious-depressive disorders *unrelated* to COVID-19	27 (32)
	Suicidal ideation *related to* COVID-19	9 (11)
	Other mental symptoms (namely: depression, psychosis, addiction, cognitive symptoms affecting memory and/or attention, psychosis, psychotic disorders, stress-related disorders)	5 (6)
Which are the most common psychiatric diagnoses among patients affected by COVID-19?	Depressive disorders	21 (25)
	Anxiety disorders	46 (54)
	Adjustment disorder	31 (36)
	Psychotic disorders	8 (9)
	Delirium	40 (47)
	Other (namely: cognitive dysfunctions, sleep disorders, substance abuse)	2 (2)
How often do you use alternative psychopharmacological strategies with patients affected by COVID-19?	Never	21 (25)
	Sometimes	47 (57)
	Often	14 (17)
	Always	1 (1)
Did you experience anxiety while consulting patients affected by COVID-19?	Yes	36 (41)
	No	51 (59)
While consulting patients affected by COVID-19, did you experience any of the following?	Anxiety	
	Yes	36 (41)
	No	51 (59)
	Depressive mood	
	Yes	8 (9)
	No	79 (91)
	Marked distress	
	Yes	16 (20)
	No	65 (80)
	Depersonalization	
	Yes	1 (1)
	No	80 (99)
	Emotional isolation	
	Yes	4 (5)
	No	77 (95)
	Hopelessness, helplessness	
	Yes	12 (14)
	No	75 (86)
	Flashbacks	
	Yes	4 (5)
	No	83 (95)
	Nightmares	
	Yes	5 (6)
	No	82 (94)
Did you need a psychological/psychiatric support in your work during this period (or after it) due to your assistance COVID-19-positive patients?	No	61 (74)
	Yes	3 (4)
	In some situations	18 (22)
Did you test positive for COVID-19?	Yes	33 (39)
	No	51 (61)
Did a colleague of yours test positive for COVID-19?	Yes	49 (69)
	No	22 (31)

### Inferential analysis

To identify specific work features associated with psychiatrists’ need for seeking help, first a binary variable was generated, starting from the results of item 16: “Did you need a psychological/psychiatric support in your work during this period (or after it) due to your assistance COVID-19-positive patients?” As Table 2 reports, 61 psychiatrists answered “no” (the 74% of the sample), while 3 answered “yes” and 18 answered “in some situations.” Therefore, the new variable was equal to 1 for all psychiatrists who reported the need for psychological/psychiatric help and was equal to 0 otherwise.

Table 3 presents the results of the univariate logistic regressions. The variables that reached a level of significance indicated by a *p*-value <0.20 were included in the multivariate regressions. Three multivariate regressions were built, one per each part of the questionnaire (Table 4). Among sociodemographic features, increased age was associated with increased risk of seeking psychological/psychiatric help by psychiatrists (OR = 1.01, *p* < 0.01), while increased years of medical experience were associated with decreased risk (OR = 0.69, *p* = 0.01). When consulting non-COVID-19 patients face-to-face, using a protective uniform was associated with decreased risk of seeking psychological/psychiatric help by psychiatrists (OR = 0.07, *p* = 0.01). Finally, when consulting COVID-19 patients, the following results were reported: psychiatric referral carried out using the internal telephone line of the hospital was associated with decreased risk of seeking help (OR = 0.03, *p* < 0.01); differently, experiencing anxiety when consulting COVID-19-positive patients was associated with increased risk of seeking help (OR = 34.06, *p* < 0.01).

**Table 3.** Results of the univariate logistic regressions

Variable	Odds ratio	*p*-Value	No. of obs.
*First part of the questionnaire: sociodemographic information*			
Age	1.10	<0.01	82
Age (squared)	1.01	<0.01	82
Gender (1 men)	1.47	0.46	82
Specialist (1) vs. resident (0)	9.05	0.04	82
Years of medical experience	1.06	0.01	82
Years of experience in the field of consultation-liaison psychiatry	1.36	0.08	76
*Second part of the questionnaire: referrals for patients not affected by COVID-19.*			
In which conditions do you assist non-COVID-19 somatic hospitalized patients? (Please, tick the correct answer/s) Face-to-face consultations using a protective uniform	0.07	0.01	81
Did the COVID-19 pandemic have an impact on the method you visit hospitalized non-COVID patients? (1 =yes, 0 = no)	0.27	0.03	81
Did your calls to see non-COVID-19 patients during this period? (1 = yes, 0 = no)	1.46	0.22	81
Did the diagnosis differ in non-COVID-19 patients during this period? (1 = yes, 0 = no)	0.41	0.42	81
*Third part of the questionnaire: referrals for patients affected by COVID-19.*			
Did you have any specialized training in the treatment of patients affected by COVID-19? (1 = yes, 0 = no)	2.83	0.08	81
In which conditions do you carry out referrals with patients affected by COVID-19? (1 = yes, 0 = no)			
a) Face-to-face consultations using protective face mask	1	1.00	76
b) Face-to-face consultations using a protective uniform	1.39	0.70	76
c) Consultations using the patient’s mobile phone	0.23	0.18	76
d) Consultations using the internal phone line in the hospital	0.10	0.03	76
e) Consultations based on the interview with the ward doctor responsible for the patient	0.07	0.01	76
What symptoms do you see most frequently among patients affected by COVID-19? (1 = yes, 0 = no)			
a) Anxiety/depressive mood related to COVID-19	0.67	0.48	76
b) Anxiety/depression mood unrelated to COVID-19	5.80	<0.01	76
c) Suicidal ideation related to COVID-19	0.84	0.84	76
d) Delirium/other mental symptoms due to COVID-19	0.42	0	76
*Which are the most common psychiatric diagnoses in this group of patients?* (1 = presence, 0 = absence)			
a) Depressive disorders	1.85	0.26	81
b) Anxiety disorders	0.35	0.04	82
c) Adjustment disorders	1.40	0.51	82
d) Psychotic disorders	1.53	0.53	82
e) Delirium	0.23	<0.01	82
In COVID-19-positive patients did you have to use alternative pharmacological strategies comparing to COVID-19 patients: 0) Never 1) Sometimes 2) Frequently 3) Always	4.07	<0.01	82
Did you experience anxiety when consulting COVID-19-positive patients? 0) Never 1) In some situations 2) Frequently 3) Always	8.20	<0.01	65
While consulting COVID-19-positive patients did you experience any of the below? (Please, tick the correct answer/s)			
a) Anxiety	0.52	0.22	80
b) Depressive mood	0.37	0.37	80
c) Marked distress	2.08	0.23	74
d) Depersonalization (if yes, predicts success perfectly)	–	–	73
e) Emotional isolation	0.96	0.98	74
f) Hopelessness, helplessness	0.22	0.16	80
g) Flashbacks	0.93	0.96	80
h) Nightmares (if yes, predicts failure perfectly)	–	–	75
Did you test positive for COVID-19? (0 = no, 1 = yes)	1.02	0.96	1.02
Did some colleague in your CLP Unit test positive for COVID-19? (0 = no, 1 = yes)	0.64	0.43	70

*Note*: Response variable: “need a psychological/psychiatric support in your work during this period (or after it) due to your assistance COVID-19-positive patients”: no = 0, yes = 1. Robust standard errors were used in all regressions. With respect to item “In which conditions do you assist non-COVID-19 somatic hospitalized patients? (Please, tick the correct answer/s),” the following answers were excluded from the analysis, since they predicted failure (outcome = 0) perfectly: consultations using protective face mask; consultations using the patient’s mobile phone; consultations using internal phone line in the hospital; consultations based on the interview with the doctor responsible for the patient. In the same way, the item “Did you see COVID-19 hospitalized patients?” was excluded since it predicted success (outcome = 1) perfectly. All these variables, having no change in their value (0 vs. 1), could not be used for the regression analysis.

**Table 4.** Results of the stepwise multivariate logistic regressions

Variable	Odds ratio	*p*-Value	No. of obs.
*Model 1 – Sociodemographic features associated with psychiatrists’ need for seeking psychological/psychiatric help*			
Age (squared)	1.01	<0.01	76
Years of medical experience	0.69	0.01	76
*Model 2* – *Features concerning working with non-COVID-19 patients associated with psychiatrists’ need for seeking psychological/psychiatric help*			
In which conditions do you carry out referrals with patients *not* affected by COVID-19? Face-to-face consultations using a protective uniform (1 = yes, 0 = no)	0.07	0.01	80
*Model 3* – *Features concerning working with COVID-19 patients associated with psychiatrists’ need for seeking psychological/psychiatric help*			
In which conditions do you carry out referrals with patients affected by COVID-19? Consultations using the internal phone line in the hospital (1 = yes, 0 = no)	0.03	<0.01	59
Did you experience anxiety when consulting COVID-19-positive patients? 0) Never 1) In some situations 2) Often 3) Always	34.06	<0.01	59

*Note*: Response variable: “need a psychological/psychiatric support in your work during this period (or after it) due to your assistance COVID-19-positive patients”: no = 0, yes = 1. Robust standard errors were used in all regressions.

## Discussion

Aim of this study was to assess the activity of psychiatrists working in the field of CLP throughout Europe during the COVID-19 pandemic and to identify work features associated with seeking psychological/psychiatric help by the psychiatrists.

As far as the impact of the pandemic on CLP activity is concerned, almost 60% of respondents reported no relevant change on the method used to carry out consultations with hospitalized non-COVID-19 patients, for whom the request of referrals remained substantially stable during the pandemic. The same can be said with respect to psychiatric diagnoses.

The only change that stemmed out concerning consultation with patients not affected by COVID-19 is represented by increased use of personal protective equipment (PPE), namely protective face mask (85% of the sample), which might have impacted on the usual way of carrying out consultation.

With respect to CLP activity involving patients affected by COVID-19, most respondents reported having not received a specialized training for the treatment of COVID-19 patients (80%). This can be due to the novelty represented by the COVID-19 virus.

While consulting patients affected by COVID-19, the most adopted PPE were protective uniform and face protective mask. The most common diagnoses were anxious-depressive disorders *related* to COVID-19, delirium due to COVID-19, and anxious-depressive disorders unrelated to COVID-19, findings consistent with previous research [2, 10, 27–33]. Differently, with respect to suicidal ideation among patients affected by COVID-19, in our sample, it was less frequent than what is reported in the literature [2, 34].

As far as the features associated with work-related stress are concerned, older age was associated with increased need to receive psychological and/or psychiatric help. This finding may be linked to the association between older age and severer clinical manifestations of COVID-19. Interestingly, this finding is not consistent with previous research carried out in other medical areas, namely ICUs, where increased risk was associated with younger age [16].

On the other hand, the protective factor represented by years of activity in the medical field stemmed out by this study is consistent with the one by Stafseth et al. [16], suggesting that more expertise in the medical field may be linked to higher ability to cope with new situations such as a pandemic.

It is worth noting that in our study the use of PPE (such as protective uniform and mask) was associated with decreased need for seeking help. This finding points out the importance of safety workplaces and the availability of PPE that may help the psychiatrist carry out his/her work [5]. Evidence suggests that when such devices are not available, the risk of psychological problems such as burn out increases [35].

With respect to the activity of CLP carried out during the pandemic involving patients affected by COVID-19, it stemmed out that the possibility of consulting using the GH internal phone line was associated with decreased probability of seeking help. During the pandemic, the possibility represented by telemedicine and tele mental health has made it possible to continue treatment even in the worst phases of the pandemic [36–39], even though some criticism was expressed, suggesting that telepsychiatry be more applicable to community mental health programs than to hospitalized patients [22]. Notably, the Guidelines from the Centers for Disease Control and Prevention recommend the use of telemedicine when possible [8]. Reducing the contact with patients was associated with reduced risk of infection, as well as decreased risk of infecting other people, such as relatives and partner.

Finally, the experience of anxiety while consulting COVID-19-positive patients was associated with increased probability of seeking psychological and/or psychiatric help. This finding suggests the possibility of pre-existing distress, subthreshold symptoms or frank disorder that deserve further investigation. On the other hand, it may be hypothesized that the availability of PPE can at least partly reduce anxiety linked to carrying out consultations with COVID-19 infected patients, and stresses once again the importance of safety workplaces.

While several studies suggested the importance of implementing targeted interventions aiming to support health care professionals and protect them from work-related stress and increased workload strictly linked to the COVID-19 pandemic [2, 25, 40, 41], the present research highlights the need of taking care of the mental health of mental health professionals, namely consultation-liaison psychiatrists. Yet, this study has several limitations that need to be acknowledged. First, its cross-sectional nature does not permit inferential conclusions. Also, the enrollment strategy may have partly influenced the results. Yet, such study design made the research feasible, and the findings of this research may be used to design future, prospective studies. The lack of a control group represents a second limit of this study. Specifically, we do not have baseline information as well as a benchmark to compare our results with (i.e., before the onset of the pandemic). A third limit may be represented by the sample size, which may have produced only a partial picture of the phenomenon investigated. Yet, this study aimed to provide descriptive hints which may help plan and design further studies. A fourth limit may be the lack of psychometric assessment for healthcare workers included in the study. Though hypothesizing that the pandemic may have increased distress perceived by psychiatrists, this variable was not directly measured, but only indirectly inferred. Such approach should be considered when assessing our findings and the study conclusions. A fifth limit in that no structured diagnostic instruments were used during the psychiatric referral, yet all psychiatric interviews were conducted by senior psychiatrists, with long experience in the field of consultation-liaison psychiatry. Moreover, when referrals were carried out by trainees, senior psychiatrists supervised them. A sixth and final limit is represented by the fact that healthcare workers may have experienced distress or psychopathological symptoms, but they could have not been consulting anyone to seek help. Further studies are needed to overcome such limitations.

To conclude, the COVID-19 pandemic seems not to have significantly changed workload and diagnoses with respect to referrals concerning patients not affected by COVID-19. Differently, among patients affected by COVID-19, the pandemic seems to have caused mainly anxiety disorders, delirium, and adjustment disorders. One psychiatrist out of four working in CLP services may have had the need for psychological/psychiatric support due to the assistance to patients affected by COVID-19. This result prompts to the need of addressing health issues of health care professionals, namely psychiatrists working in the field of CLP and psychosomatics.

## Data Availability

The data that support the findings will be available upon request to the corresponding author following a 36-month embargo from the date of publication to allow further steps concerning this study.
